# CD8 T-cell-mediated protection against liver-stage malaria: lessons from a mouse model

**DOI:** 10.3389/fmicb.2014.00272

**Published:** 2014-06-06

**Authors:** Natalija Van Braeckel-Budimir, John T. Harty

**Affiliations:** Department of Microbiology, University of IowaIowa, IA, USA

**Keywords:** CD8 T cells, memory, protection, *Plasmodium*, mice, humans

## Abstract

Malaria is a major global health problem, with severe mortality in children living in sub-Saharan Africa, and there is currently no licensed, effective vaccine. However, vaccine-induced protection from *Plasmodium* infection, the causative agent of malaria, was established for humans in small clinical trials and for rodents in the 1960s. Soon after, a critical role for memory CD8 T cells in vaccine-induced protection against *Plasmodium* liver-stage infection was established in rodent models and is assumed to apply to humans. However, these seminal early studies have led to only modest advances over the ensuing years in our understanding the basic features of memory CD8 T cells required for protection against liver-stage *Plasmodium* infection, an issue which has likely impeded the development of effective vaccines for humans. Given the ethical and practical limitations in gaining mechanistic insight from human vaccine and challenge studies, animal models still have an important role in dissecting the basic parameters underlying memory CD8 T-cell immunity to *Plasmodium*. Here, we will highlight recent data from our own work in the mouse model of *Plasmodium* infection that identify quantitative and qualitative features of protective memory CD8 T-cell responses. Finally, these lessons will be discussed in the context of recent findings from clinical trials of vaccine-induced protection in controlled human challenge models.

## INTRODUCTION

Malaria represents an enormous global health problem. It is associated with around 200 million reported annual cases and more than 600,000 deaths, most of them recorded in sub-Saharan Africa (WHO, 2011). Current disease treatment is limited to antimalarial drugs targeting the symptomatic blood-stage infection. Given the enormous genetic plasticity of the parasite, the emergence of antimalarial drug resistance is inevitable and thus a major concern (Miller et al., 2013). Hence, the development of a protective malaria vaccine is alarmingly urgent.

Blocking *Plasmodium* infection at the level of the silent, liver stage of malaria represents an attractive strategy for disease prevention (Kappe et al., 2010). The earliest evidence of vaccine-induced, sterile, liver-stage immunity originates from mouse studies, in which it was demonstrated that vaccination with radiation attenuated sporozoites (RAS) prevented development of blood-stage *Plasmodium berghei* infection after sporozoite challenge (Nussenzweig et al., 1967, 1969). Importantly, RAS-induced sterile protection was confirmed in human subjects, in whom it was induced upon exposure to bites of more than 1000 sporozoite-bearing, irradiated mosquitoes (Clyde et al., 1973; Edelman et al., 1993; Hoffman et al., 2002). Persistent efforts to repeat the success of RAS in inducing sterilizing immunity using different vaccine formulations and regimens (e.g., which are easier to manufacture and can be administered through approved vaccination routes) have led only to a partial success (Moorthy et al., 2004; Dunachie et al., 2006; Bejon et al., 2007; Moorthy and Ballou, 2009; Agnandji et al., 2012). Therefore, RAS immunization still represents the gold standard for induction of sterile protection and, despite logistical challenges, has moved recently to Phase I clinical trial.

Besides being a potential vaccine candidate, RAS has been used as an invaluable tool for studying protective immune responses against liver-stage *Plasmodium* infections. Although the earliest work describes neutralizing antibodies as the main mechanism of RAS-induced protection (Potocnjak et al., 1980; Yoshida et al., 1980), more recently depletion studies and adoptive-transfer experiments have demonstrated that CD8 T cells have a leading role in protection against sporozoite challenge (Schofield et al., 1987; Weiss et al., 1988; Rodrigues et al., 1991). The critical role of CD8 T cells in protection against liver-stage malaria has been confirmed upon immunization with different formulations, such as recombinant vaccines [e.g., *Salmonella typhimurium*, vaccinia virus or adeno virus expressing *P. berghei* circumsporozoite (CS) protein] and genetically attenuated parasite (GAP; Sadoff et al., 1988; Lanar et al., 1996; Rodrigues et al., 1997; Jobe et al., 2007).

Despite the strong evidence for the role of CD8 T cells in sterile protection against malaria, critical qualitative and quantitative characteristics of the protective response and effector mechanisms engaged by CD8 T cells remain incompletely understood. The modest progress in this field is strongly influenced by the extreme genetic plasticity of the parasite, its complex life cycle and the paucity of defined antigenic targets for CD8 T cells. Filling these knowledge gaps is of an utmost importance, as this information would facilitate the development of successful pre-erythrocytic vaccine candidates.

Although highly desirable, studies of *Plasmodium* infection in humans are limited by multiple ethical and practical factors (inability to manipulate the immune response for mechanistic studies, difficulty to access relevant samples, etc.). Therefore, progress in understanding immunity against liver-stage malaria critically depends on the availability of suitable animal models. Infection of mice with *P. berghei* and *Plasmodium yoelii*, two rodent *Plasmodium* pathogens, closely resembles the early stages of human liver invasion, replication, and development within hepatocytes (Meis et al., 1983; Sturm et al., 2006; Baer et al., 2007). Additionally, clear differences in infectivity and pathogenicity displayed by these two *Plasmodium* species mimic the diversity of human *Plasmodium* infections (Sedegah et al., 2007). Therefore, the mouse model has proven to be invaluable for basic studies, such as host–parasite interactions and the underpinning of the immune mechanisms driving protection induced upon vaccination and sporozoite inoculation.

Here, we will present and highlight lessons about protective memory CD8 T-cell thresholds for liver-stage protection and effector mechanisms engaged by these cells, learned from vaccinations of mice with both subunit (*Plasmodium* CS-derived epitope) and whole-parasite (RAS and GAP) vaccine formulations. Finally, we will discuss the outcomes of recent vaccine clinical trials in light of our own findings and highlight the implications of the lessons learned for further development of liver-stage malaria vaccines.

## INDUCTION OF CD8 T CELLS BY SUBUNIT VACCINES

### SETTING THE NUMERIC THRESHOLD

Induction of sterilizing immunity against liver-stage parasite represents a challenge for at least two reasons. First, liver-residing or recruited CD8 T cells have to locate and eliminate all the infected hepatocytes/parasites to prevent progression of the infection from the liver to the blood stage. A single mosquito bite delivers a few hundred infectious sporozoites into the skin dermis. Given that only a fraction of sporozoites actually reaches the liver and infects hepatocytes, we estimate that only 1 out of 10^9^ hepatocytes in humans or 1 out of 10^6^ hepatocytes in mice are infected after mosquito infection. Similarly, low frequencies of infected hepatocytes are likely after intravenous (i.v.) challenge with 100–1000 virulent sporozoites, as used in many mouse studies. Thus, surveying CD8 T cells target extremely rare events, creating the proverbial “needle in a haystack” scenario. Second, the time interval between the start of the liver-stage infection and release of blood-stage merozoites is very short (7 days in *Plasmodium falciparum* and 2 days in *P. berghei/P. yoelii*), which means that CD8 T cells have a limited amount of time to perform their task (Sturm et al., 2006; Sturm and Heussler, 2007; Todryk and Hill, 2007). In the context of these spatial and temporal pressures, it is important to gain knowledge about the quantitative and qualitative features of protective memory CD8 T-cell response.

To address these questions, we used a mouse model to induce a stable, long-lasting memory CD8 T-cell response against a defined epitope (*P. berghei* CS_252__-__26__0_). For this purpose, we exploited an accelerated prime-boost approach that is well established in our laboratory (Badovinac et al., 2005). In short, mice were vaccinated with mature dendritic cells (DCs), which had been incubated for 2 h with CS-derived peptide to allow surface peptide–MHC complexes to form (DC-CS), and a week later they received a booster vaccination with an attenuated *Listeria monocytogenes* expressing the same CS-derived peptide (LM-CS; Schmidt et al., 2008). This vaccination strategy (from here on abbreviated as DC-LM) has proven to be a robust tool for the generation and study of stable, long-lasting memory CD8 T-cell response against defined antigenic determinants without contribution by other components of the immune response (CD4 T cells, antibodies, NK cells; Schofield et al., 1987; Weiss et al., 1993; Doolan and Hoffman, 1999, 2000).

This vaccination approach allowed us to induce CS-specific CD8 T-cell immune responses with a magnitude of 1–7% of the total peripheral blood leukocytes (PBLs) (up to 20% of CD8 T cells). Strikingly, this response was stable and protective against repeated sporozoite challenges for at least 19 months (the life span of a laboratory mouse). Moreover, by titrating the booster vaccine dose, we were able to induce CD8 T-cell response with decreasing magnitude, which allowed us to determine the potential numeric threshold required for protection. Strikingly, we observed that more than 95% of animals with CD8+ T-cell frequencies exceeding a threshold of 1% of total PBLs were protected against sporozoite challenge, while more than 95% of animals with CD8 T-cell frequencies below this threshold developed blood-stage infection and were thus not sterilely protected (Schmidt et al., 2008). These findings demonstrate that sterile protection against sporozoite infection requires a remarkably strong CD8 T-cell response, representing a substantial fraction of the total CD8 T-cell pool and highly exceeding frequencies of antigen-specific CD8 T cells required for plausible protection against various viral and bacterial infections (Schmidt et al., 2008). On the other hand, given the previously mentioned spatial and temporal pressures on the sterilizing CD8 T-cell-mediated response, these results do not come as a complete surprise. It is not difficult to imagine that recognition and elimination of all the rare infected hepatocytes within 2 days require mobilization of extremely high CD8 T-cell numbers.

Thus, we describe a quantitative feature of protective memory CD8 T-cell response against liver-stage *Plasmodium*, and show that if met, this feature can potentially ensure life-long protection.

### DISSECTING EFFECTOR FUNCTIONS UTILIZED BY PROTECTIVE MEMORY CD8 T CELLS

Various effector molecules, such as IFN-γ, TNF-α, perforin, FasL, and TRAIL, are utilized by memory CD8 T cells in protection against different infections (Raeder et al., 2000; Trapani and Smyth, 2002; Shrestha et al., 2006, 2008; Ishikawa et al., 2009). A few attempts were made to define the effector component of the CD8 T-cell responses against liver-stage *Plasmodium* infections. As these studies were based on RAS immunizations, which in addition to CD8 T-cell responses also induce also CD4 T cell and antibody responses, it is still not completely clear which pathways are engaged by memory CD8 T cells (Ferreira et al., 1986; Schofield et al., 1987; Tsuji et al., 1995; Renggli et al., 1997; Rodrigues et al., 2000). Furthermore, it is not clear whether CD8 T-cell responses against different *Plasmodium* species require the same effector pathways for sterile immunity. This information is of high relevance for the development of human vaccines, which would ideally protect against multiple *Plasmodium* species.

To study effector functions in well-defined memory CD8 T-cell population, we used the DC-LM prime/boost approach. In contrast to vaccination with whole-parasite formulations, which in addition to CD8 T cells elicit also non-CD8 T-cell responses, this immunization approach allows focus only on effector pathways utilized by memory CD8 T cells (Schmidt et al., 2009). Memory CD8 T-cell responses against *P. beghei* and *P. yoelii* CS-derived peptides were induced in wild-type (wt) BALB/c mice together with mice deficient for various effector molecules (IFN-γ, perforin, FasL, and TRAIL). Additionally, TNF-α was depleted by neutralizing antibodies in vaccinated, wt mice to assess its role in CD8 T-cell-mediated sterile protection.

The most important finding of the study was that the pathways of memory CD8 T-cell-mediated protection against liver-stage infection were not completely overlapping for the two different *Plasmodium* species (Butler et al., 2010). Protection against *P. berghei* was diminished in the absence of IFN-γ and TNF-α but was not influenced by the absence of perforin. In contrast, the absence of perforin, but not TRAIL and FasL, completely eliminated protection against *P. yoelii*. In line with this finding, induction of generalized inflammation by treatment of animals with TLR9 agonist (CpG) 24 h-post sporozoite infection was sufficient to block the progression of *P. berghei*, but not *P. yoelii* infection to the blood stage. Susceptibility of *P. yoelii* sporozoites to CpG-induced inflammation was observed only during a very short window of 12 h-post infection. As *P. yoelii* displays higher infectivity in rodents compared to *P. berghei* (Sedegah et al., 2007), it is likely that more stringent control of parasite replication and development, involving direct killing of infected hepatocytes through the perforin pathway, are required for successful control of this infection at the liver stage.

Thus, effector mechanisms exploited by memory CD8 T cells in protection against liver-stage infection are *Plasmodium* species specific, a finding of high relevance for development of protective human vaccine targeting clinically relevant *P. falciparum* and *P. vivax*.

### INFLUENCE OF *Plasmodium*–HOST INTERACTIONS ON MEMORY CD8 T-CELL-MEDIATED PROTECTION AGAINST LIVER-STAGE *Plasmodium* INFECTION

Although we demonstrated a clear threshold (>1% of PBL) for CD8 T-cell-mediated sterilizing immunity, our findings were limited to one mouse strain (BALB/c) and one *Plasmodium* species (*P. bergei*; Schmidt et al., 2008). The observation that some mouse strains, e.g., C57Bl/6 and B10.D2, are more difficult to protect against sporozoite infection upon RAS vaccination than BALB/c (Weiss et al., 1989; Doolan and Hoffman, 2000) made us wonder to what extent the protective threshold is influenced by host–parasite interactions defined by host-strain-specific background genes. Multiple studies have acknowledged the important role of non-MHC-linked host background genes on development of an immune response against infections (Hsieh et al., 1995; Diosi, 2002). Of equal importance, human malaria is caused by infection with multiple *Plasmodium* species, thus raising the question to what extent the protective threshold is influenced by different parasite species.

In our hands, following the DC-LM prime/boost with *P. yoelii* CS_280__-__288_, mice developed high-magnitude memory CD8 T-cell responses that exceeded the defined threshold established with *P. berghei* model (>1% of total PBL). Interestingly, unlike our observations after *P. berghei* infection, these animals were highly susceptible even to a low-dose, *P. yoelii* challenge (Schmidt et al., 2011). Although we were able to substantially increase the size of memory CD8 T-cell population (>1.5% of total PBL), we were unable to find a defined, numerical threshold required for stable (defined as >80%) protection against liver-stage infection with *P. yoelii*. This observation was in line with the finding that protection against *P. yoelii* infection requires more stringent control of parasite replication, achieved by direct killing of infected hepatocytes by engagement of the perforin pathway (Butler et al., 2010). Thus, the protective memory CD8 T-cell threshold was highly influenced by the species of *Plasmodium* even in a single mouse strain.

**FIGURE 1:**
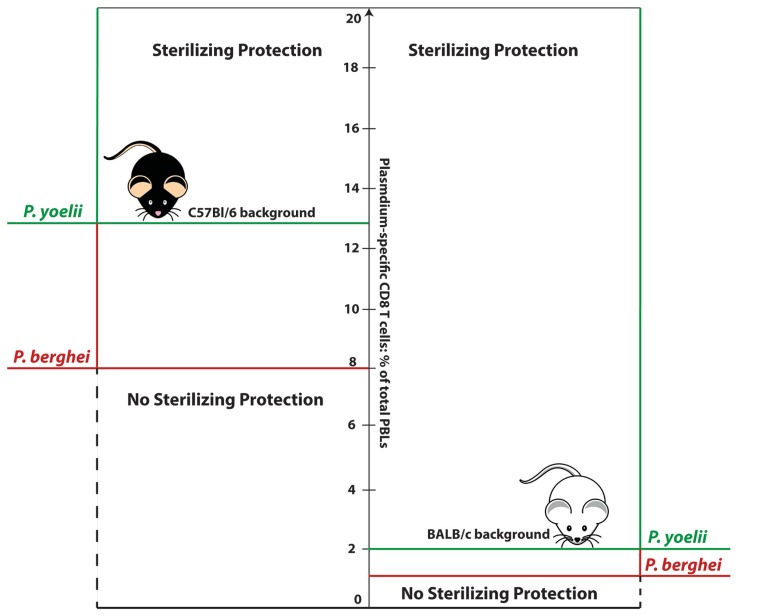
**Numerical thresholds required for memory CD8 T-cell-mediated sterilizing protection from sporozoite challenge: influence of host background genes and *Plasmodium* strain.** Sterilizing protection of BALB/c mice against *P. berghei or P. yoelii* requires numbers of antigen- (CS-derived epitope) specific memory CD8 T-cell equivalent to ~1% or 2% of total PBLs, respectively. Sterilizing protection of mice with C57Bl/6 background requires substantially higher CD8 T-cell numbers: ~8% of total PBLs in the case of *P. berghei* infection, or ~13% in the case of *P. yoelii* infection.

In parallel with this, we observed that different strains of mice expressing the same MHC class I molecule (H-2K^d^) that presents the CS-epitopes, but differing in background genes, display dramatically different levels of susceptibility to *P. berghei* sporozoite challenge (**Figure 1**). Importantly, in all these mouse strains, the DC-CS/LM-CS vaccination induced memory CD8 T-cell responses of similar magnitude and quality (e.g., production of IFN-γ; Schmidt et al., 2011). While more than 80% of immunized BALB/c and DBA/2 mice were protected against the infection, protection observed in mice with C57Bl/6 or closely related C57Bl/10 background genes (CB6F1 and B10.D2, respectively) was marginal or completely absent. Only after administration of a second booster vaccination dose, which induced extremely high-magnitude memory CD8 T-cell response (~15% of the total PBL or ~60% of the total CD8+ T cells), were B10.D2 and CB6F1 mice protected from sporozoite challenge. Furthermore, a study performed on reciprocal bone marrow chimeras between B10.D2 and BALB/c mice revealed that B10.D2 T-cell immune response reconstituted on a BALB/c background displays a superior protection in comparison to BALB/c T-cell response reconstituted on C57Bl/10 background. All the evidence results suggest that it is not the functional or quantitative property of memory CD8 T-cell response, but rather the host–pathogen interactions, determined by the host-background gene milieu and parasite species, that are key factors determining thresholds for memory CD8 T-cell-mediated protection. Of note, we have not identified a mouse background that is easier to protect against *Plasmodium* sporozoite infection.

This knowledge obtained in a mouse malaria model is of a particular relevance for understanding differences in protection observed in heterogeneous human populations and for development of successful malaria vaccines, which have to ensure protection against multiple, human *Plasmodium* species. Given the modest success of current human vaccines in inducing CD8 T-cell response, the extremely high numerical requirements for sterile protection against malaria may be an additional challenge for vaccine development.

### INFLUENCE OF UNRELATED INFECTIONS ON MAINTENANCE OF *Plasmodium*-SPECIFIC MEMORY CD8 T CELLS AND PROTECTION

In the parts of the world where malaria is endemic, infections with various, malaria-unrelated pathogens are also very common (Corbett et al., 2006; Magambo et al., 2006; Hotez and Kamath, 2009). Therefore, it is very likely that the burden of multiple infections has a substantial impact on the maintenance of both numbers and quality of *Plasmodium*-specific memory CD8 T cells, the major mediators of protection against liver-stage infection (Selin et al., 1996, 1999; Welsh and Selin, 2009). The phenomenon of attrition of antigen-specific memory CD8 T-cell populations has been described earlier in the context of infections with unrelated viruses (Selin et al., 1996, 1999). Specifically, these authors showed that antigen-specific memory CD8 T cells induced in mice by exposure to a viral infection underwent a decrease in frequency and numbers upon subsequent infection with unrelated viruses.

Investigating to which extent memory CD8 T cells are affected by unrelated infections and whether this potential effect can be reversed is of high relevance as it may provide basic information about development and reshaping of *Plasmodium*-specific immune response and useful guidelines for development of successful vaccine regimens.

In a separate study, we showed that exposure of mice to infections with unrelated pathogens (e.g., LCMV, LM, VacV, MHV-1) following DC-LM CS prime/boost vaccination induces dramatic decreases in frequency, but also in total numbers of antigen-specific memory CD8 T cells (Schmidt and Harty, 2011). Furthermore, more pronounced overall attrition was observed in the subpopulation of effector memory (Tem) compared to central memory (Tcm) T cells. This change in subset composition may have substantial functional consequences, as we previously suggested that Tem, but not Tcm are closely correlated with protection against sporozoite challenge (Schmidt et al., 2010). These drastic changes in numbers and composition of antigen-specific memory CD8 T-cell subsets resulted in seriously compromised protection, as only 10% of immunized mice exposed to malaria-unrelated infections were protected against sporozoite challenge. Importantly, the induced attrition did not hamper the capacity of memory CD8 T cells to expand upon antigen re-encounter. In line with this, a single-booster immunization after virus-induced attrition induced expansion of the memory population and restored the subpopulation structure (Tem vs. Tcm), and, most importantly, the protection against sporozoite challenge.

As multiple pathogens are endemic to the same geographic areas as malaria, it is very likely that vaccination-induced memory CD8 T-cell response will undergo a certain degree of attrition, which poses an additional concern for successful vaccination. The finding that despite the attrition, memory CD8 T cells remain responsive to booster vaccination, suggests that the use of prime and regular booster vaccination approach may be critical to preserve long-term immunity against malaria.

## INDUCTION OF CD8 T CELLS BY WHOLE-PARASITE VACCINES

### BROADENING OF THE ANTIGEN REPERTOIRE BY VACCINATION WITH RAS: IMPACT ON NUMERICAL THRESHOLDS

To date, only two immunization approaches have demonstrated a capacity to induce sterile protection against liver-stage malaria in both humans and animal models. Recently, an immunization approach based on multiple, low-dose inoculations of wt sporozoites through mosquito bites in individuals were also continually treated with the antimalarial drug chloroquine drug was evaluated. This approach was shown to successfully protect humans from a subsequent mosquito bit challenge (Roestenberg et al., 2011). The second approach is RAS vaccination, which still represents the gold standard of antimalarial protection (Clyde et al., 1973; Edelman et al., 1993; Hoffman et al., 2002). Nevertheless, critical parameters of protection, such as quantity and quality of the RAS-induced memory CD8 T-cell remain unknown.

After establishing that sterile immunity against sporozoite challenge in mice vaccinated with a single *Plasmodium* CS epitope requires mobilization of a large portion of total memory CD8+ T-cell pool (Schmidt et al., 2008), we decided to probe the hypothesis that broadening the antigenic targets would decrease the threshold of memory CD8 T cells required for protection. Multiple, low-magnitude, immune responses against broad range of antigenic determinants may have a superior protective capacity in comparison to monospecific response. For this purpose, the RAS vaccination regime was applied (Schmidt et al., 2010). To overcome the lack of well-defined MHC class I-restricted antigenic determinants, we took advantage of a surrogate activation marker approach recently described by our laboratory for measuring CD8 T-cell responses to bacterial or viral pathogens (Rai et al., 2009). Activation of effector or memory antigen-specific CD8 T cells by exposure to viral or bacterial infection induces down regulation of CD8α and up-regulation of CD11a. Importantly, these changes are stable in well-defined, antigen-specific CD8 T cells for the life of the laboratory mouse. This approach allowed us for the first time to follow and quantitatively and qualitatively characterize RAS-specific memory CD8 T-cell response and to assess the induced protection in multiple mouse strains.

To our surprise, broadening of the CD8 T-cell antigenic repertoire by vaccination with RAS did not measurably decrease the numerical threshold of memory cells required for protection (Schmidt et al., 2010). As shown by specific antibody depletion studies, the protection depended on CD8 T cells but not CD4 T cells (Schmidt et al., 2010). Similarly to our results with CD8 T cells specific for CS-derived epitopes, sterile protection was highly dependent on the mouse strain and *Plasmodium* species (**Figure 1**). Thus, BALB/c mice were relatively “easy” to protect, as a single dose (20,000) of RAS was sufficient to induce sterile protection against *P. berghei* challenge in > 80% of animals, and a single-booster immunization ensured protection against highly virulent *P. yoelii* challenge. On the other hand, protection observed in C57Bl/6 mice after a single RAS immunization was absent, despite similar memory CD8 T-cell numbers induced upon vaccination in both mouse strains. Solid, sterile protection of these mice against *P. berghei* sporozoite challenge required multiple booster doses and multiple-fold increase in the magnitude of the CD8 T-cell response. Antigen-specific CD8 T-cell populations representing even 40% of total CD8 T cells in B6 mice were not sufficient to protect against *P. yoelii* challenge at a memory time point.

Importantly, application of surrogate activation markers allowed us for the first time to follow and characterize RAS-specific memory CD8 T-cell responses and subsequent protection in populations of outbred mice, which may be a better model for genetically diverse human populations. In striking contrast to more or less uniform memory CD8 T-cell responses induced in inbred mice, responses measured in outbred groups varied substantially in magnitude and kinetics among individual animals. Despite wide distribution of immune responses, >80% of prime-boosted outbred mice were sterilely protected against homologous challenge with *P. berghei* and *P. yoelii* sporozoite infection. Importantly, this finding could potentially predict the distribution of immune responses upon whole-parasite immunization at the level of heterogeneous human populations.

### MULTIPLE BENEFITS FROM TARGETING LATE-LIVER-STAGE ANTIGENS

Recently, a new sporozoite attenuation strategy has been developed, based on a direct manipulation (deletion) of target parasite genes (Mueller et al., 2005b). Such a targeted attenuation method may be superior in comparison to irradiation. While irradiation induces random lesions in DNA molecules and consequently gives rise to genetically diverse population of RAS, genetic manipulation allows for controlled production of well-defined and genetically similar parasites (genetically attenuated parasites – GAP; Chattopadhyay et al., 2009; Kappe et al., 2010). Additionally, while attenuation by irradiation arrests the parasite development at an early liver stage (Menard et al., 2013), targeting specific genes crucial for various metabolic processes allows for more or less custom design of GAP and production of both early- and late-liver-stage-arresting parasites (Mueller et al., 2005a,b; Aly et al., 2008). GAP have been tested in multiple pre-clinical studies and induced CD8 T-cell-mediated protection in murine malaria models (Labaied et al., 2007; Tarun et al., 2007).

Given the different degrees of intrahepatic development between early-liver-stage-arrested RAS and GAP and late-liver-stage-arrested GAP, we hypothesized that the latter expresses a broader spectrum of antigen determinants, and possibly induces superior protection. Consistent with this notion, vaccination with late-liver-stage-arrested GAP induced higher memory CD8 T-cell responses, which closely correlated with better protection induced in BALB/c, but also C57Bl/6 and Swiss Webster mice (Butler et al., 2011). Importantly, the antigenic specificity of the memory CD8 T-cell immune responses induced by early- and late-liver-stage-arresting vaccines was only partially overlapping. From our study, it was clear that late-liver-stage-arresting GAP induce responses against broader spectrum of antigens compared to both RAS and early-liver-stage-arrested GAP. Thus, we concluded that superior protection induced upon vaccination with late-liver-stage-arrested GAP was based on the broadening of the antigenic repertoire.

These findings are particularly important in the light of recent finding of Tarun et al. (2008), who predicted that late-liver-stage and blood-stage parasites have substantial overlap in their antigen pools. This would mean that vaccination with late-liver-stage-arrested GAP may also induce cross-stage protection. Indeed, mice vaccinated with high numbers of late-liver-stage-arrested GAP, but not RAS, displayed high degree of protection against both liver- and blood-stage challenge (Butler et al., 2011).

Targeting a broad spectrum of antigens, particularly the ones shared by different developmental stages of the parasite, may increase the protective efficacy of a vaccine (**Table 1**). Not only do late-liver-stage-arrested GAP represent a promising vaccine candidate, but these parasites may also serve as an important model for determining novel CD8 T-cell antigens, which might be exploited for design of new generation of cross-protective subunit vaccines.

**Table 1:** Comparison between early- and late-liver stage arresting whole-sporozoite vaccines.

Vaccine	Mode of inactivation	Stage of arrest	Percentage of sterilizing protection after a single dose	Protection against blood-stage challenge	Cross-species protection
RAS	Irradiation	Early liver	5	No	Partial
*sap1*^-^ GAP	Mutagenesis	Early liver	20	No	–
*fabb*^-^ GAP	Mutagenesis	Late liver	40	Yes	Yes

## OUTCOME OF RECENT HUMAN TRIALS: NUMBERS DO MATTER

Although a substantial body of evidence has been collected pointing to the relevance of CD8 T-cell response in controlling liver-stage malaria, current vaccines have displayed rather modest capacity of inducing such responses in humans. The most advanced malaria vaccine is RTS,S, a subunit vaccine based on CS protein, which induces partial protection that correlates with antibody and CD4 T-cell immune responses, but no detectable CD8 T-cell response (Moorthy and Ballou, 2009). In the most recent field clinical trial in children this vaccine displayed ~30% efficacy against clinical malaria in the target population of 6–12-month-old infants (Agnandji et al., 2012). Thus, there is a need for development of new vaccine candidates with the capacity to elicit CD8 T-cell response, and which can be used alone or in combination with RTS,S.

One of the promising CD8 T-cell-inducing approaches is based on heterologous prime-boost strategies using different viral vectors expressing the same CD8 T-cell-target antigens (Hill et al., 2010). Unfortunately, most of the vaccination regimens that were successful in mice failed when tested in humans (Ockenhouse et al., 1998; Moorthy et al., 2004; Bejon et al., 2006, 2007). Recently, a new promising prime-boost regimen based on chimpanzee adenovirus/MVA expressing *Plasmodium* thrombospondin adhesive protein (TRAP) fused to a multiple epitopes derived from several malaria antigens has been successfully tested in pre-clinical studies (Colloca et al., 2012), followed by two clinical trials in humans (O’Hara et al., 2012; Ewer et al., 2013). Results of the Phase IIa clinical trial revealed that 20% of vaccinated subjects had sterilizing immunity against standardized sporozoite infection, and 35% of vaccines displayed significant delay to a patency, which represent an additional measure of vaccine efficacy (Ewer et al., 2013). Although the degree of protection was modest, a very important finding of the study was the close correlation between the observed protection and the increased percentage of total CD8 T cells producing vaccine antigen-specific IFN-γ, over that induced in previous vaccine trials. Moreover, the efficiency of protection (sterile protection vs. delay to a patency vs. lack of protection) was dependent on the magnitude of IFN-γ-producing CD8 T cells, suggesting the existence of protective threshold, which is in line with our finding in a mouse model. The existence of protective CD8 T-cell thresholds may explain the failure of previous vaccination regimens tested in humans (Schmidt et al., 2008, 2010). On the other hand, it remains possible that protective memory CD8 T-cell thresholds in humans may be lower (<0.3% of total CD8 T cells) than those we observed in mice (>15% of total CD8 T cells). One of the possible explanations is the prolonged intra-hepatic phase of sporozoite infection in humans (6–8 days), compared to mice (2 days), providing human CD8 T cells with substantially more time for detection and elimination of infected cells, which might decrease the cell numbers required for successful protection.

Another recent human trial confirmed the importance of magnitude of CD8 T-cell response in protection against liver-stage malaria. Although it has been known for four decades that vaccination of human subjects with RAS via mosquito bites induces sterile protection against subsequent sporozoite challenge, only recently the first aseptic, radiation-attenuated, metabolically active, purified, cryopreserved, sporozoite vaccine was tested in a clinical trial, when it was injected into human subjects via two standard vaccination routes: subcutaneous (s.c.) and intradermal (i.d.; Epstein et al., 2011). Interestingly, the result of this first RAS clinical trial was the absence of protection in all the subjects, regardless of the dose received. With the hypothesis that the failure in protection was due to the suboptimal vaccination route rather than to lack of vaccine immunogenicity, Seder et al. (2013) performed another clinical trial using the intravenous (i.v.) route for the vaccine delivery. The hypothesis was initially tested and confirmed in both non-human primate and a mouse model, as which revealed that i.v. immunization with RAS induces superior protection against sporozoite challenge when compared to s.c. immunization (Epstein et al., 2011). Using the previously described surrogate activation markers to enumerate antigen-specific CD8 T cells, authors showed that i.v. immunization induces immune response of substantially higher magnitude in liver and spleen than s.c. immunization. In line with these findings, and in striking contrast to s.c. vaccination, i.v. vaccination of human subjects induced sterilizing protection in 80% of subjects receiving the highest vaccine dose. Moreover, the protection correlated with numbers of CD8 T cells producing IFN-γ in a majority of protected subjects. These findings demonstrate superior protection induced by i.v. administration of RAS, and suggest induction of sufficient numbers of antigen-specific CD8 T cells as underlying mechanism of the observed protection.

Together these two studies suggest the translational value of our main finding in a mouse malaria model: the importance of memory CD8 T-cell numbers in sterilizing protection against liver-stage malaria and the potential existence of a definable threshold for sterilizing immunity. Besides being fundamental findings of high importance for further improvement of current and development of new malaria vaccine candidates and vaccination regimens, these findings also justify the use of a mouse as a suitable system for studying basic principles of immunity against pre-erythrocytic malaria stage.

## CONCLUDING REMARKS

In summary, recent information generated in the mouse model of *Plasmodium* infection sets the cornerstone for further research of CD8 T-cell-mediated protection against liver-stage malaria. The most striking finding is that induction of extremely high numbers of memory CD8 T cells is a prerequisite for solid, sterile protection. Therefore, developing vaccines or vaccination regimens that will ensure induction of high-magnitude memory CD8 T-cell response against selected, highly protective target antigens is one means for successful immunization. However, it is still not known whether induction of additional immune components, such as CD4 T cells and antibodies, would positively influence the protection, for example, by decreasing the required numbers of CD8 T cells. Additionally, induction of CD8 T cells specific for antigens shared by (late) liver-stage and blood-stage parasite is a promising approach toward cross-stage, and possibly cross-species protection. Thus, one focus for future research should be discovery and characterization of such “shared” epitopes. Finally, the precise qualitative features leading to optimal memory CD8 T-cell protection against liver-stage *Plasmodium* infection remain to be determined.

## Conflict of Interest Statement

The authors declare that the research was conducted in the absence of any commercial or financial relationships that could be construed as a potential conflict of interest.
